# Supplementary material for: Exposure to Phthalate and Organophosphate Esters via Indoor Dust and PM10 Is a Cause of Concern for the Exposed Saudi Population
Source: Int J Environ Res Public Health. 2021 Feb 22;18(4):2125. doi: 10.3390/ijerph18042125 (PMC7926922; doi:10.3390/ijerph18042125)
Supplement: Supplementary file 1 [file ijerph-18-02125-s001.pdf]

Supplementary Information

Exposure to Phthalate and Organophosphate esters via Indoor dust and PM10 is a Cause of Concern for the Exposed Saudi Population

Nadeem Ali[1]*, Nabil A. Alhakamy[2], Iqbal M.I. Ismail[1,3], Ehtisham Nazar[4], Ahmed Saleh Summan[1,5], Syed Ali Musstjab Akbar Shah Eqani[6], Govindan Malarvannan[7] *

[1]Centre of Excellence in Environmental Studies, King Abdulaziz University, Jeddah 21589, Saudi Arabia
[2]Pharmaceutics department, Faculty of Pharmacy, King Abdulaziz University, Jeddah 21589, Saudi Arabia
[3]Department of Chemistry, Faculty of Science, King Abdulaziz University, Jeddah 21413, Saudi Arabia
[4]Department of Environmental Sciences, University of Gujrat, Punjab 50700, Pakistan
[5]Department of Environmental Sciences, King Abdul Aziz University, Jeddah 21589, Saudi Arabia
[6]Public Health and Environment division, Department of Biosciences, COMSATS Institute of Information Technology, Islamabad 45550, Pakistan
[7] Toxicological Centre, University of Antwerp, Universiteitsplein 1, Wilrijk 2610, Belgium

Corresponding Authors
***Dr. Nadeem Ali**
Center of Excellence in Environmental Studies, King Abdulaziz University
P.O Box: 80216 Jeddah 21589, Saudi Arabia
Fax: +966(12)6951674; Phone: +966(12)6402000 Ext: 20777
E-mail: nabahadar@kau.edu.sa ; chakrian2010@gmail.com

Dr. Govindan Malarvannan, PhD
Toxicological Center | University of Antwerp
Campus Drie Eiken | Building S, 5th Floor
Universiteitsplein 1 | 2610 Wilrijk | Belgium
Tel: +32-3-265 2743 | Fax: +32-3-265 2722
E-mail: malarvannan.govindan@uantwerpen.be

This supplementary information consists of four tables.

Table S1. Mean levels (µg/g of dust) of selected OPEs and phthalates in SRM 2585.

**Compounds**	**van den Eede et al. [1]**	**Brandsma et al. [2]**	**Luongo et al. [3]**	**Mercier et al. [4]**	**Bergh et al. [5]**	**This study**
TCEP	0.70	0.80	1.1			0.80
TCPP	0.90	0.80	1.3			1.2
TDCPP	3.2	2.5	2.6			2.7
TPhP	1.2	0.90	1.5			1.5
DMP			0.60	2.5	1.0	2.1
DEP			5.2	8.2	6.7	8.5
DBP			38	33	31	37
DEHA						0.70
DZBP			85	99	93	111
DEHP			538	552	570	612

Table S2. Concentrations (ng/m^3) of analyzed OPEs and phthalates in indoor air reported from different countries.

Countries	Microenvironment	n	TCEP	TCPP	TDCPP	TPhP	DMP	DEP	DBP	DEHA	DZBP	DEHP	Reference
Saudi Arabia	Kitchen	15	6	43	<LOQ	15	<LOQ	9	191	14	1	610	This study
	Hotel	10	4	42	<LOQ	7	<LOQ	62	255	6	1	230	
	Living room	20	7	38	<LOQ	9	<LOQ	11	250	10	1	520	
	Office	10	<LOQ	12	<LOQ	3	<LOQ	28	173	4	2	109	
Sweden	Daycare	11	25	8.4	<LOQ	<LOQ	4.7	870	600	nd	21	240	Bergh et al. [6]
	Home	10	4.8	5.6	<LOQ	<LOQ	15	1300	850	nd	21	200	
	Work	10	10	100	28	<LOQ	4.4	620	550	nd	15	100	
Germany	Day care	63	<2	2.7	<LOQ	nd	nd	nd	nd	nd	nd	nd	Fromme et al. [7]
Germany	Home	7	<0.2	4.15	<0.1	<0.16	nd	nd	nd	nd	nd	nd	Zhou et al. [8]
	Office	11	0.56	33.9	0.42	<0.16	nd	nd	nd	nd	nd	nd	
	Day care	6	1.7	6.49	3	<0.16	nd	nd	nd	nd	nd	nd	
	School	12	<0.2	10	<0.1	<0.16	nd	nd	nd	nd	nd	nd	
	Store	9	<0.2	20.6	<0.1	<0.16	nd	nd	nd	nd	nd	nd	
Norway	Home	48	2.25	42.3	0.084	nd	nd	nd	nd	nd	nd	nd	Cequier et al. [9]
Japan	Home	6	13-65	nd	<LOQ-18	<LOQ-78	<LOQ-210	10-440	100-4000	3-420	5.0-72	31-2400	Takeuchi et al. [10]
China	Office	90	1-13.5	0.8-81	0.63	0.3-10	nd	nd	nd	nd	nd	nd	Yang et al. [11]
Finland	Office	4	8	20	<40	<6	nd	nd	nd	nd	nd	nd	Makinen et al. [12]
	School	3	100	160	<40	40	nd	nd	nd	nd	nd	nd	
	Store	7	20	<10	<40	9	nd	nd	nd	nd	nd	nd	
Switzerland	Office	4	26.5	nd-130	nd	nd	nd	nd	nd	nd	nd	nd	Hartmann et al. [13]
	Store	2	9.1	51.1	nd	nd	nd	nd	nd	nd	nd	nd	
USA	Multi-indoor settings	54	2.24	17.3	1.51	1.11	nd	nd	nd	nd	nd	nd	Kim et al. [14]

Table S3. Concentrations and range (ng/g) of dust OPEs in indoor dust reported from different countries.

Countries	n	Year	TCEP	TCPP	TPhP	TDCPP	References
Saudi Arabia	House, n=20	2019	280<br><LOQ–25450	1280<br>26–17250	795<br>610–9000	1525<br><LOQ–	This study
	AC filter, n=15		14475<br><LOQ-58190	1750<br>670-5830	920<br>790-2520	5950<br>630-19660	
	House, n=20		<LOQ<br><LOQ-3370	2650<br>150-7820	835<br>670-1540	660<br>400-5630	
Saudi Arabia	Mosque, n=30	2017	420<br>270-34700	2055<br>1570-4820	1135<br>675-4960	2490<br>970-6945	Ali et al. [15]
Egypt	20	2014	22<br><8–132	28<br><10–123	67<br>8–289	72<br><9–557	Abdallah and Covaci [16]
Kuwait	15	2011	710<br>275–1800	1460<br>120–7065	430<br>44–6890	360<br>60–1555	Ali et al. [17]
Pakistan	15	2011	15<br><10–175	<20<br><20–85	175<br><2–330	<5<br><5–255	Ali et al. [17]
Japan	40	2006	7500<br><1300–	18700<br>5400–	5400<br><1600–	4000<br><1200–	Kanazawa et al. [18]
USA	16	2011	2700<br>330–110000	2200<br>490–	2800<br>790–36000	2100<br>920–44000	Dodson et al.[19]
New Zealand	34	2010	110<br>20–7650	350<br>20–7620	565<br>20–7500	230<br>20–16550	Ali et al.[20]
Belgium	15	2010	590<br><80–5460	2940<br>580–24400	1970<br>150–34200	760<br><80–56200	Van den Eede et al. [1]
Sweden	10	2010	2100<br><80–33000	1600<br>700–11000	1200<br>100–4200	10000<br>2200–27000	Bergh et al. [6]
Romania	47	2010	100<br><20–1160	860<br><20–16400	500<br><20–22600	60<br><20–460	Dirtu et al. [21]
Spain	8	2007	510<br>250–9800	3800<br>350–10300	1850<br>290–9500	125<br><LOQ–1100	García et al. [22]
Germany	6	2006	200<br>140–280	740<br>370–960	380<br>180–1300	<80<br><80–110	Brommer et al. [23]

Table S4. Concentrations (ng/g) of dust phthalates in indoor dust reported from different countries.

Country	n	DMP	DEP	DBP	BzBP	DEHP	Reference
Saudi Arabia	20	0.29	1.02	18.85	0.46	573	This study floor dust
	10	0.41	4.15	41.45	0.75	745	Hotel dust
	20	0.34	1.14	26.70	0.93	672	AC filter floor
Saudi Arabia	15	0.6	1.4	33	0.8	1022	Albar et al. [24]
Kuwait	15	0.1	2.7	2	1	240	
Sweden	62	0.47	14	103	16	449	Luongo et al. [3]
Sweden	346	–	–	150	135	770	Bornehag et al. [25]
Denmark	497	–	1.7	15	3.7	210	Langer et al. [26]
Germany	30	1.5	6.1	47	30	703	Fromme et al. [7]
Germany	30	–	–	87	15	604	Abb et al. [27]
France	7	0.35	4.9	9.1	6.1	462	Mercier et al. [4]
Kuwait	21	0.02	1.8	45	7.9	2256	Gevao et al. [28]
Bulgaria	177	280	340	9930	340	1050	Kolarik et al. [29]
China	215	0.1	0.2	24	1.6	183	Zhang et al. [30]
China	75	0.2	0.4	20	0.2	228	Guo and Kannan [31]
USA	33	0.08	2	13	21	304	
USA	120	–	5	20	45	340	Rudel et al. [32]
France	1	–	44	22	9.3	185	Santillo et al. [33]
Spain	1	0.4	9.5	120	142	195	
UK	29	0.12	12	50	57	192	
Finland	3	1.3	85	49	32	354	
Denmark	3	–	0.7	34	26	179	
Sweden	2	–	11	62	79	223	

References

(1) N. Van den Eede, A.C. Dirtu, H. Neels, A. Covaci, Analytical developments and preliminary assessment of human exposure to organophosphate flame retardants from indoor dust. Environ. Int. **2011**, 37, 454–461.

(2) S.H. Brandsma, J. de Boer, M.J. van Velzen, P.E. Leonards, Organophosphorus flame retardants (PFRs) and plasticizers in house and car dust and the influence of electronic equipment. *Chemosphere*, **2014,** *116*, 3-9.

(3) G. Luongo C. Östman Organophosphate and phthalate esters in settled dust from apartment buildings in Stockholm. *Indoor Air.* **2015,** 26, 414-425.

(4) F. Mercier, E. Gilles G. Saramito P. Glorennec, B. Le Bot, A multi-residue method for the simultaneous analysis in indoor dust of several classes of semi-volatile organic compounds by pressurized liquid extraction and gas chromatography/tandem mass spectrometry. *J Chromatogr A.* **2014,** 1336,101–111.

(5) C. Bergh, G. Luongo, S. Wise, C. Östman, Organophosphate and phthalate esters in standard reference material 2585 organic contaminants in house dust. *Anal. Bioanal. Chem*. **2012,** *402,* 51-59.

(6) C. Bergh, R. Torgrip, G. Emenius, C. Östman, Organophosphate and phthalate esters in air and settled dust–a multi-location indoor study. *Indoor air*, **2011,** *21,* 67-76.

(7) H. Fromme, T. Lahrz, M. Kraft, L. Fembacher, C. Mach, S. Dietrich, R. Burkardt, W. Völkel, T. Göen, Organophosphate flame retardants and plasticizers in the air and dust in German daycare centers and human biomonitoring in visiting children (LUPE 3). Environ. Int. **2014,** *71*, 158-163.

(8) L. Zhou, M. Hiltscher, D. Gruber, W. Püttmann, Organophosphate flame retardants in indoor and outdoor air in the Rhine/Main area, Germany: comparison of concentrations and distribution profiles in different microenvironments. *Environm Sci Pollut Res*, **2017,** *24*, 10992-1100.

(9) E. Cequier, A.C. Ionas, A. Covaci, R.M. Marcé, G. Becher, C. Thomsen, Occurrence of a broad range of legacy and emerging flame retardants in indoor environments in Norway. *Environ Sci Technol.* **2014,** *48,* 6827-6835.

(10) S. Takeuchi, H. Kojima, I. Saito, K. Jin, S. Kobayashi, T. Tanaka-Kagawa, H. Jinno, Detection of 34 plasticizers and 25 flame retardants in indoor air from houses in Sapporo, Japan. Sci. Total Environ, **2014**, *491*, 28-33.

(11) F. Yang, J. Ding, W. Huang, W. Xie, W. Liu, Particle size-specific distributions and preliminary exposure assessments of organophosphate flame retardants in office air particulate matter. *Environ Sci Technol.* **2013,** *48,* 63-70.

(12) M.S. Mäkinen, M.R. Mäkinen, J.T. Koistinen, A.L. Pasanen, P.O. Pasanen, P.J. Kalliokoski, A.M. Korpi, Respiratory and dermal exposure to organophosphorus flame retardants and tetrabromobisphenol A at five work environments. *Environ Sci Technol.* **2009,** *43,* 941-947.

(13) P.C. Hartmann, D. Burgi, W. Giger, Organophosphate flame retardants and plasticizers in indoor air. Chemosphere **2004,** 57, 81–787.

(14) U.J. Kim, Y. Wang, W. Li, K. Kannan, Occurrence of and human exposure to organophosphate flame retardants/plasticizers in indoor air and dust from various microenvironments in the United States. Environ. Int. **2019,** *125,* 342-349.

(15) N. Ali, I.M.I. Ismail, M.W. Kadi, H.M.S.A. Albar, Currently used organophosphate flame retardants determined in the settled dust of masjids and hotels of Saudi Arabia, a new insight into human health implications of dust exposure. *Environ Sci: Process & Impacts*, **2018,** *20,* 798-805.

(16) M.A.E. Abdallah, A. Covaci, Organophosphate flame retardants in indoor dust from Egypt: implications for human exposure. *Environ Sci Technol.* **2014,** *48,* 4782-4789.

(17) N. Ali, L. Ali, T. Mehdi, A.C. Dirtu, F. Al-Shammari, H. Neels, A. Covaci, Levels and profiles of organochlorines and flame retardants in car and house dust from Kuwait and Pakistan: implication for human exposure via dust ingestion. Environ. Int. **2013,** *55,* 62-70.

(18) A. Kanazawa, I. Saito, A. Araki, M. Takeda, M. Ma, Y. Saijo, R. Kishi, Association between indoor exposure to semi–volatile organic compounds and building–related symptoms among the occupants of residential dwellings, Indoor Air, **2010**, 20, 72–84.

(19) R.E. Dodson, L.J. Perovich, A. Covaci, N. Van den Eede, A.C. Ionas, A.C. Dirtu, J.G. Brody, R.A. Rudel, After the PBDE phase-out: a broad suite of flame retardants in repeat house dust samples from California. *Environ Sci Technol.* **2012,** *46,* 13056-13066.

(20) N. Ali, A.C. Dirtu, N. Van den Eede, E. Goosey, S. Harrad, H. Neels, A. Mannetje, J. Coakley, J. Douwes, A. Covaci, Occurrence of alternative flame retardants in indoor dust from New Zealand: indoor source and human exposure assessment. Chemosphere **2012,** 88, 1276–1282.

(21) A.C. Dirtu, N. Ali, N. Van den Eede, H. Neels, A. Covaci, Country specific comparison of profile of chlorinated, brominated and other phosphate organic contaminants in indoor dust. Case study for Eastern Romania 2010. *Environ Int.* **2012**, 49, 1–8.

(22) M. Garcia, I. Rodriguez, R. Cela, Microwave-assisted extraction of organophosphate flame retardants and plasticizers from indoor dust samples. *J Chromatogr A.* **2007,** *1152,* 280-286.

(23) S. Brommer, S. Harrad, N. Van den Eede, A. Covaci, Concentrations of organophosphate esters and brominated flame retardants in German indoor dust samples. J. Environ. Monit. **2012**, 14, 2482–2487.

(24) H.M.S.A. Albar, N. Ali, K. Shahzad, I.M.I. Ismail, M.I. Rashid, W. Wang, L.N. Ali, S.A.M.A.S. Eqani, Phthalate esters in settled dust of different indoor microenvironments; source of non-dietary human exposure. *Microchem J.* **2017,** *132,* 227-232.

(25) C.G. Bornehag, B. Lundgren, C.J. Weschle, T. Sigsgaard, L. Hagerhed-Engman, J. Sundell Phthalates in indoor dust and their association with building characteristics. *Environ Health Persp.* **2005,** 113, 1399-1404.

(26) S. Langer, G. Bekö, C.J. Weschler, L.M. Brive, J. Toftum, M. Callesen, G. Clausen, Phthalate metabolites in urine samples from Danish children and correlations with phthalates in dust samples from their homes and daycare centers. *Int J Hyg Environ health.* **2014,** 217, 78-87.

(27) M. Abb, T. Heinrich, E. Sorkau, W. Lorenz, Phthalates in house dust. *Environ Int.* **2009,** 35, 965-970.

(28) B. Gevao, A.N. Al-Ghadban, M. Bahloul, S. Uddin, J. Zafar, Phthalates in indoor dust in Kuwait: Implications for non-dietary human exposure. *Indoor Air.* **2013,** 23,126-133.

(29) B. Kolarik, C.G. Bornehag, K. Naydenov, J. Sundell, P. Stavova, O.F. Nielsen, The concentrations of phthalates in settled dust in Bulgarian homes in relation to building characteristic and cleaning habits in the family. *Atmos. Environ.* **2008,** *42,* 8553-8559.

(30) Q. Zhang, X.M. Lu, X.L. Zhang, Y.G. Sun, D.M. Zhu, B.L. Wang, R.Z. Zhao, Z.D. Zhang, Levels of phthalate esters in settled house dust from urban dwellings with young children in Nanjing, China. *Atmos Environ.* **2013,** 69, 258-264.

(31) Y. Guo, K. Kannan, Comparative assessment of human exposure to phthalate esters from house dust in China and the United States. *Environ Sci Technol.* **2011,** *45,* 3788-3794.

(32) R.A. Rudel, D.E. Camann, J.D. Spengler, L.R. Korn, J.G. Brody, Phthalates, alkylphenols, pesticides, polybrominated diphenyl ethers, and other endocrine-disrupting compounds in indoor air and dust. *Environ Sci Technol.* **2003**, 37, 4543-4553.

(33) D. Santillo, I. Labunska, H. Davidson, P. Johnston, M. Strutt, O. Knowles, *Consuming chemicals: Hazardous chemicals in house dust as an indicator of chemical exposure in the home*. Greenpeace Research Laboratories, Department of Biological Sciences, University of Exeter: Exeter, UK, 2003.
